# Attachment- and Relationship-Based Interventions during NICU Hospitalization for Families with Preterm/Low-Birth Weight Infants: A Systematic Review of RCT Data

**DOI:** 10.3390/ijerph19031126

**Published:** 2022-01-20

**Authors:** Soo-Yeon Kim, Ah Rim Kim

**Affiliations:** 1 Department of Nursing, Daegu Haany University, Gyeongsan City 712-715, Korea; sooyeonkim@dhu.ac.kr; 2 Department of Nursing, Far East University, 76-32 Daehak-gil, Gamgok-myeon, Eumseong-gun 27601, Korea

**Keywords:** attachment, preterm infant, systematic review, interventions, hospitalizations

## Abstract

This study conducts a systematic review and meta-analysis of the randomized-controlled clinical trials (RCTs) of attachment- and relationship-based interventions in the NICU. A systematic search of the PubMed, MEDLINE, Embase (OVID), PsycINFO, and CINAHL databases and the Cochrane Database of Systematic Reviews was conducted in February 2021. Of the 32,904 studies examined, 15 were identified as relevant, and 10 RCTs were eligible for meta-analysis. Cochrane’s risk of bias tool was used to assess the quality of the trial reporting. Interventions were categorized as (1) parent–infant interactions, (2) parent education, and/or (3) support through qualitative synthesis. The attachment- or relationship-based intervention was effective in relieving maternal traumatic stress, maternal depression, infant weight growth, and infant development. Subgroup analyses suggested that interventions significantly improved sub-domains of mothers’ and children’s interactive behavior. Tailored, staged interventions may contribute to better health outcomes in preterm infants and their families.

## 1. Introduction

The formation of strong attachments is fundamental to optimal growth and brain development in the first two years of life; this is a complex biological process influenced by the environment and interpersonal relationships [1,2]. The concepts describing the early parent–infant relationship, such as “attachment,” “bonding,” and “attachment bonding,” have been used interchangeably throughout various disciplines, although they involve essentially distinctive theories and processes. For example, “attachment” describes how the relationship toward an attachment figure—or, specifically, the primary caregiver—is built and developed from the child’s perspective. In contrast, bonding focuses on the parent’s affective, cognitive, and behavioral manifestation of feelings, views, or actions toward the infant [3,4]. Compared to full-term children, preterm infants or children born before 32 weeks of gestational age (GA) or weighing less than 1500 g and with subsequent hospitalization in the neonatal intensive care unit (NICU), experience lower secure attachment rates [5]. Newborns admitted to the NICU have been reported to develop attachment disorders, such as disorganized attachment, at rates approximately six times higher at 36 months of age [6]. Thus, NICU environmental and interpersonal factors should be considered potential risks for attachment insecurity, regardless of infants’ medical vulnerability due to preterm birth.

Infants hospitalized in the NICU, as well as their families, face traumatic experiences and challenging environments that might create parental psychosocial stress, such as early separation between parents and infants, altered or disrupted parental roles, artificial lights and noises, inevitable invasive medical procedures, and a lack of contact and interaction in parent–infant dyads [7,8]. Therefore, the process of establishing a relationship in the NICU requires a different perspective [3], as parent–infant relationships or attachment may suffer from a lack of parental competence and involvement in infant care; less closeness and proximity; and parental psychiatric vulnerabilities, such as post-traumatic stress, depression, or anxiety [7]. Once at-risk families are identified prenatally and beyond discharge, multidisciplinary family-centered interventions in diverse NICUs focusing on optimal infant–parent relationships would be important in facilitating family health and infant developmental outcomes [7,8].

Hence, this study defined attachment- and relationship-based interventions as NICU interventions intended to improve attachments, bonding, and relationships in parent–infant dyads. These include components of parent–infant interactions, such as play, parental proximity, or sensitivity to infant cues; attachment-oriented programs, such as skin-to-skin contact or kangaroo care; parenting or caregiving practices; and participation in infant care.

Well-designed randomized-controlled clinical trials (RCTs) could benefit infant and family outcomes, as they could facilitate parental bonding and proximity to the infant and enhance the parental role and parent–infant interaction during NICU hospitalizations. Literature in the last five years has included a systematic review with qualitative synthesis of published papers on attachment among the NICU population [9], systematic reviews of early parenting intervention in populations of young children [10], a study of a population younger than 13 [11], and observations of at-risk families in the first year of infancy [12]. However, the effectiveness of interventions focused on promoting attachment between mothers and preterm infants during NICU hospitalization has seldom been comprehensively examined.

Our systematic review synthesizes findings from RCTs published in the past 22 years, or from 1999 to the present. This is because a new perspective emerged in 1999 regarding the attachment process in mother–infant pairs to illustrate this as an individualized process rather than merely a natural one [4,13]. The current review aims to synthesize the key components of attachment- and relationship-based interventions for preterm infants and their families in the NICU. Further, it will determine their effects on mother–infant bonding, attachments, and relationships; parental psychosocial or mental health; and infants’ growth and developmental outcomes.

## 2. Methods

A systematic literature review and meta-analysis were conducted to investigate the effects of attachment- and relationship-based interventions for preterm infants and their families on attachments, relationships, and parental psychosocial or infant health-related outcomes. We adhered to a previously published plan of investigation as outlined in our study protocol (Systematic Review Registration: PROSPERO CRD42019145834) [4]. 

### 2.1. Core Questions

A key question was set in accordance with the PICO-SD (participants/population, intervention, comparison, outcomes, and study design) strategy. (1) Participants/Population (P): preterm infants (GA < 37 weeks or birth weight < 2500 g); (2) Intervention (I): attachment/bonding- and relationship-based interventions, including components of parent–infant interaction, parent education, and support; (3) Comparison (C): the typical care group or no-treatment, different intervention group; (4) Outcome (O): attachment/relationship outcomes, such as the quality of parent–infant interactions, maternal postpartum attachment, and parental bonding; parental psychosocial outcomes, such as anxiety, stress, or depression; and infant growth and developmental outcomes, such as neuro-developmental, behavioral, or cognitive-emotional issues, or body weight or physiological conditions; and (5) Study Design (SD): in this study, the use of RCTs.

### 2.2. Search Strategy and Study Selection

**Figure 1:**
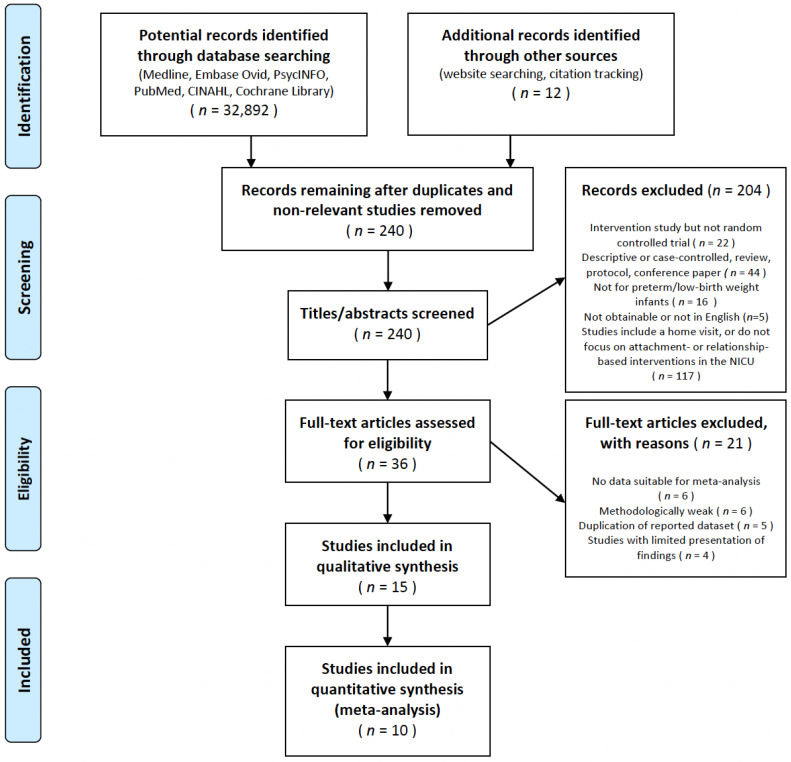
Study selection flow diagram.

Data were collected from 1 November 2020 to 1 February 2021, to analyze the effectiveness of attachments, bonding, or relationship-based interventions in NICUs. Relevant studies were sought using MeSH/Thesaurus terms in the following electronic databases: PubMed, MEDLINE, Embase (OVID), Scopus, CINAHL, the Cochrane Database of Systematic Reviews, PsycINFO (OVID), the Cochrane Central Register of Controlled Trials (CENTRAL), and Web of Science. We eliminated any publication bias by gathering available evidence regarding our topic, including such “gray” literature sources as ProQuest, the Dissertations and Theses database, OpenGREY, the Gray Literature Report, and Google Scholar. These searches included target keywords and subject headings related to attachment- or relationship-based interventions in NICUs [4]. For example, CINAHL Plus was searched using: (infant, newborn OR newborns or neonates or infants) OR (newborn, neonate, infant, or baby) OR (premature infants or preterm infants or premature baby or preterm baby) OR (low birth weight or small for gestational age or low weight) OR VLBW OR LBW, Newborn or infan *, or neonate *. The two authors conducted each step in the study selection process using the Preferred Reporting Items for Systematic Reviews and Meta-Analyses (PRISMA) flowchart [14]. An adapted PRISMA flowchart revealed the inclusion and exclusion processes (Figure 1). In instances of disagreement among the reviewers, a discussion was held until an agreement was reached during the screening process.

### 2.3. Quality Assessment of the Selected Studies

The Cochrane collaboration tool, or Risk of Bias (RoB) 2.0 [15] was used to assess the risk of bias in RCTs. The tool consists of a randomization process, deviations from the intended intervention, missing outcome data, measurements of the outcome, and a selection of the reported results. These five domains will be comprehensively evaluated as “overall biases”; each signaling question for each area can be scored using a checklist as either “yes,” “probably yes,” “probably no,” “no,” and “no information.” This is then evaluated as “low,” “some concern,” or “high” depending on the algorithm. The two researchers independently evaluated RoB 2.0 and discussed any differing evaluation results; a final evaluation was completed after reaching an agreement.

### 2.4. Data Analysis

The characteristics of the studies included in the review were analyzed, and data were extracted based on the study title, author, publication year, study design, number of centers included, the study’s country of origin, sample size (including the number of enrolled infants and the experimental/comparative group allocation), description of the sample, the intervention information (name, deliverer, duration, and number of sessions), evaluation timing (follow-up), and the outcome variables. A meta-analysis of the Cochrane Collaboration was conducted using RevMan version 5.4 (Cochrane Collaboration, London, UK). to measure the effect size and homogeneity of the interventions in the selected studies. The heterogeneity among studies was assessed using Higgins I2 statistics with the following criteria: (1) 0%, or no heterogeneity; (2) 0% to 40%, or unimportant heterogeneity; (3) 30% to 60%, or moderate heterogeneity; and (4) 70% to 100%, or significant heterogeneity [16]. This study uses such outcome variables as maternal anxiety level, parental environmental stress, maternal traumatic stress, depression, infant weight gain, and development with heterogeneity ranging from 0% to 60%. These were analyzed using a fixed-effects model. Variables with a significant heterogeneity of greater than 70%, including parent-to-infant attachment (bonding), were analyzed using a random-effects model. When heterogeneity was too substantial to be explained, a subgroup or sensitivity analysis was employed, and qualitative synthesis was considered. Forest plots were used to visualize pooled estimates of the effect size and confidence interval (CI). Further, the effect size value was represented by the standardized mean difference across different measures of the same construct (e.g., maternal traumatic stress, parent-to-infant attachment, or bonding) and mean difference in measures of the maternal anxiety, depression, and quality of mother–infant interaction subscales. The effect sizes were statistically significant at 0.05, and the CI was set at 95%. A funnel plot and Egger’s regression test were considered when the meta-analysis included more than 10 studies due to low test power [17].

## 3. Results

### 3.1. Description of Studies

#### 3.1.1. Search Results

This review sought to identify attachment- and relationship-based intervention studies that included any assessment of attachment or relationship, NICU infant, or parental psychosocial outcomes. From 32,904 initial records, primary literature yielded 238 titles and abstracts; of these, 34 were identified by the two authors as potentially relevant for full-text review. This search was conducted until 28 February 2021 and yielded 15 studies that were identified as relevant and included in this review. The records were excluded based on the criteria presented in Figure 1.

#### 3.1.2. Study Characteristics

Table 1 presents these studies’ characteristics. Although the search was conducted for the years between 1999 and 2021, the studies included ranged from 2001 to 2021. Of the 15 included studies, research was conducted in the following countries: the United States: four (26.7%) studies; the United Kingdom: two (13.3%) studies; Taiwan: two (13.3%) studies; and one each for India, Switzerland, the Netherlands, Sweden, Australia, South Korea, and Ireland (6.6%). Seven studies were published from 2001 to 2010, while eight studies were published over 10 years (2011 to 2021). Regarding the number of samples, seven studies (46.6%) had 70 or fewer participants, four (26.7%) had 71 to 140 participants, and four (26.7%) had 141 to 210 participants. The average number of centers included in the studies was 2.2 ± 1.85, with a range of 1 to 7. All studies included preterm infants in one or two intervention groups, Groups 1 and 2, respectively [17,18], versus a control or comparison group. All studies except one included a full-term reference group [19]. Seven studies evaluated follow-up outcomes for preterm infants at different stages: at discharge and at corrected ages of 1, 3, 6, and 12 months.

#### 3.1.3. Intervention Participants

All studies involved interventions that included mother–infant pairs or dyads; however, 11 of the 15 studies measured attachment/bonding or interaction and relationship outcomes [17,18,19,22,23,24,25,26,29,30,31]. Among these, four reported the effects of interventions on maternal postpartum attachment or mother–infant bonding [22,26,29,30]. Three studies included both parents in their interventions, contributing to an understanding of the differential effects of interventions on parents’ attachment or bonding [26,30] and parenting stress [27]. Preterm infants who were born at a gestational age of less than 37 weeks and/or weighing less than 2500 g and admitted to the NICU were eligible for inclusion in most studies, while five studies [24,25,29,31] targeted extremely preterm infants, or those born earlier than 32 weeks’ gestation. One study [26] included infants born at 28 weeks’ gestational age or less, and two studies [18,29] included very low-birth weight infants, or those weighing less than 1500 g. The studies’ inclusion of multiple births differed. Mörelius et al. [27] included healthy women who delivered a single preterm infant with a gestational age of between 32 and 35 weeks. Samra et al. [28] controlled for multiple gestations by excluding multiples from the analysis, while Glazebrook et al. [24] adequately controlled for twins by clustering.

#### 3.1.4. Quality Assessment of the Evidence

**Figure 2:**
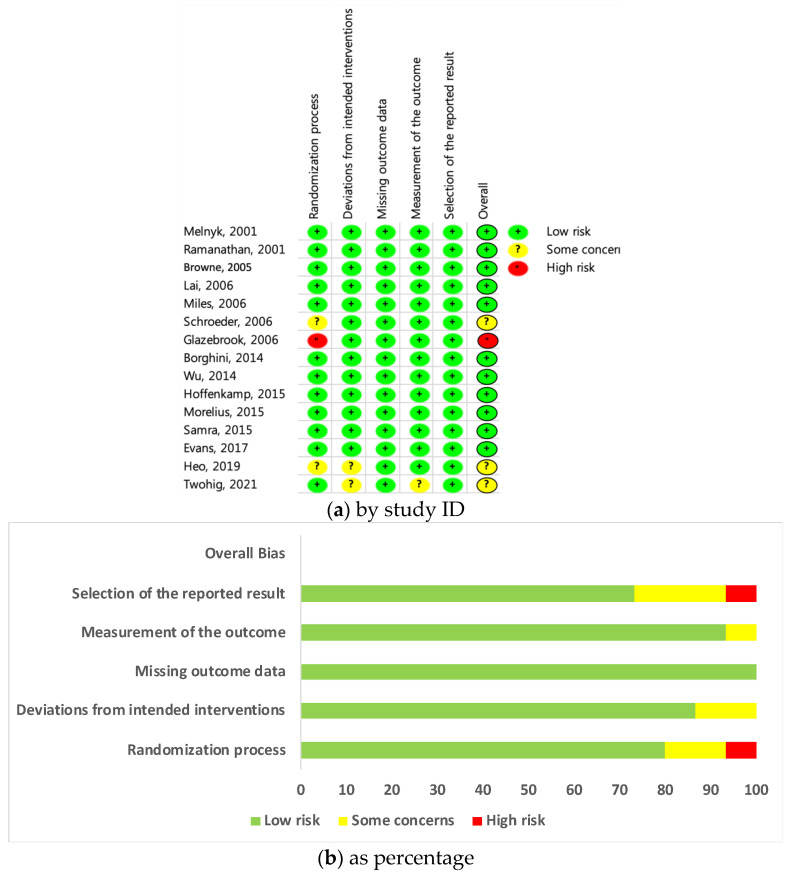
(**a**) Risk of bias assessment- by study ID; (**b**) Risk of bias assessment- by percentage.

Cochrane’s RoB 2.0 tool was used (Figure 2a) to evaluate the quality of the 15 studies [15]. The per-protocol (PP) evaluation was conducted according to the research plan to determine the effects of attachment- and relationship-based interventions (Figure 2b). Therefore, the risk of bias in the studies was assessed by focusing on the effects of intervention adherence. Of the 15 studies, one was classified as having a high risk of bias, three had some concerns, and the others were classified as low risk. This tool consists of a total of five components (randomization process; deviation from intended interventions; missing outcome data; measurement of the outcome; selection of the reported result), and each component area is a checklist that answers 3 to 7 questions in an algorithmic manner. If one or more of the five components is evaluated as “high risk,” it is ultimately evaluated as “high risk” in the overall bias. Subsequently, the work by Glazebrook et al. [24] was omitted from the meta-analysis given the identification of high risk of bias. Glazebrook et al. [24] was evaluated as “high risk” in the allocation sequence in the random process. Since this study is a cluster-randomized controlled trial, and the experimental and control groups were determined by tossing a coin, it was difficult to judge that the allocation sequence was concealed until participants were enrolled and assigned to an intervention.

The evidence for judgment was as follows. Regarding the randomization process, participants in the multi-center study assigned one center to the experimental group and another to the control group through a coin toss which was judged as an inadequate concealment of the allocation sequence, resulting in an assessment of a high risk. The other two studies did not provide information on the concealment of the allocation sequence, and one study was evaluated as having “some” concerns. Other studies comprehensively described the randomization process and concealment of allocation, and it was confirmed that the baseline was balanced. In terms of “deviations from intended interventions,” four studies mentioned that blinding was not practical or possible, or that the study could not be blinded to the researcher or participants. Other studies did not clearly describe the participant’s or assessor’s blinding. However, due to the intervention study’s nature, it was posited that it would have been difficult for the participants (parents) to be blinded, and if the participant had only one baby, the results would not have been affected even if not blinded. The important non-protocol interventions were balanced across intervention groups in 13 studies, regardless of whether the intervention was blind; therefore, it was evaluated as low risk. However, the two studies were evaluated as a concern because they did not mention important non-protocol interventions.

In terms of “missing outcome data,” all studies were judged as low risk, with thorough descriptions of the reason for any omissions. Further, sufficient data were presented regarding those studies’ results.

In terms of “measuring the outcome,” one study was judged as having some concerns because the author in this case stated that the mothers assigned to the control group could create some adverse effects as they became aware of additional interventions in the experimental group. The other studies were judged as low risk.

In terms of “selecting the reported result,” all studies were judged as low risk, as those studies appropriately reported the measurement method, timing, and analysis.

#### 3.1.5. Intervention Components

The studies provided some description of the intervention program components, delivery, and duration, except for two, which contained no comments on intervention deliverers. Parents (mothers or mother–father dyads) and nurses were the primary deliverers of interventions in six studies (40%), while the others had clinical experts or staff, such as practitioners, therapists, nurses, certified video interaction guidance professionals, and child psychiatrists. All studies had control (standard/routine/usual care) and comparison groups (e.g., informal discussions, no interaction guidance, maintaining skin-to-skin contact, or clinical- or home-based intervention programs).

Based on Benzies et al. [32], who suggested three types of intervention approaches within a bio-ecological framework, we coded intervention components into five categories: (a) parent–infant interactions; (b) parent education—only information given (E1); (c) parent education—guided observations, including parent observations or demonstrations of an activity (E2); (d) parent education—parents’ active involvement/engagement, self-evaluation, or self-reflection through video interaction guidance (E3); and (e) parent/family support tailored to a parent’s circumstances to address any socio-emotional/psychological concerns, such as social support, counseling, or consulting. The two authors assessed the intervention components reported in each study (Supplementary Materials Table S1).

All studies encouraged parents to interact with their infants—such as through free play; kangaroo care or skin-to-skin contact; talking to, feeding, smiling, or hugging the infant; or changing diapers—or to participate in a test of their behavioral reactions in responding to their infant’s cues. Three studies focused only on supporting parent–infant interactions [20,21,22]. Two studies used only information given [27,28]. The most commonly included component was parent–infant interaction, and 12 of the 15 RCT studies provided some form of education based on parent–infant interactions and/or parental support [17,18,19,22,24,25,26,27,28,30,31,32]. Two studies combined guided observation with active involvement in parent education [23,25]. Two studies used all three types of parenting education [30,31]. Only Glazebrook et al. [24] combined information with guided observation; two other studies combined information and active involvement [19,26].

### 3.2. Synthesis of Results

A meta-analysis was performed with a total of 10 papers classified into 8 variables (Supplementary Materials Table S2): two studies of attachment/bonding [22,29], two studies of mother-infant interactions [25,31], two studies of maternal anxiety [19,22], two studies of parental environmental stress [19,28], two studies of traumatic stress [25,26], two studies of maternal depression [22,26], two studies of infant weight growth [28,30], and three studies of infant development [18,19,31].

#### 3.2.1. Effects of Interventions on Attachment/Bonding

Of the 10 included studies, 4 used measured parent-to-infant attachment or parental bonding. The homogeneity test revealed Q (chi-square) = 181.86, df = 3 (*p* < 0.00001), and I2 = 98%. The results for the overall effects of interventions on attachment or bonding were insignificant. A sensitivity analysis was conducted with two of the four studies [22,29] that did not include parental education components, but measured maternal postpartum attachment with the Maternal Postnatal Attachment Scale (MPAS). This indicated that attachment- or relationship-based interventions did not significantly promote maternal postpartum attachments (*p* = 0.19) (Supplementary Materials Figure S1).

#### 3.2.2. Effects of Interventions on Mother–Infant Interactions

Two studies used the CARE-Index [25,31] to measure mother–infant interactions, including subscale scores and mothers’ and children’s interactive behaviors. The pooled effect sizes for mothers’ interactive behavior (MD = 0.06, 95% CI = −0.06–0.18, *p* = 0.30) and children’s interactive behavior (MD = 0.08, 95% CI = −0.02–0.18, *p* = 0.13) were inconclusive. These indicate the three domains of mothers’ interactive behavior; the difference in maternal control (Z = 4.39, *p* < 0.0001) and unresponsiveness (Z = 2.91, *p* = 0.004) between the two groups were statistically significant. Regarding the four domains of children’s interactive behavior, the difference in infant compliance (Z = 4.06, *p* < 0.0001), infant difficulty (Z = 13.23, *p* < 0.00001), and infant passivity (Z = 5.91, *p* < 0.00001) between the two groups were statistically significant.

#### 3.2.3. Effects of Interventions on Anxiety

Two studies [19,22] reported maternal anxiety using the State-Trait Anxiety Inventory (STAI) in the intervention and comparison groups participating in attachment- or relationship-based programs; a homogeneity test of these studies revealed Q (chi-square) = 0.00, df = 1 (*p* = 0.95), and I2 = 0%. The results for the overall effects of interventions on maternal anxiety were insignificant.

#### 3.2.4. Effects of Interventions on Parental Environmental Stress

Two studies used the CARE-Index [19,28] to measure parental environmental stress in four dimensions in the NICU: its sights and sounds, infants’ behavior and appearance, parental role alteration, and staff behaviors and communication. The pooled effect sizes for parental environmental stress revealed no significant difference between the two groups; further, the four dimensions also did not significantly differ between the two groups.

#### 3.2.5. Effects of Interventions on Maternal Traumatic Stress

Two studies [25,26] measured maternal traumatic stress symptoms, each using a different instrument. A homogeneity test revealed Q (chi-square) = 0.44, df = 1 (*p* = 0.508), and I2 = 0%. The pooled effect sizes for maternal traumatic stress (SMD = −0.33, 95% CI = −0.61 to −0.06, *p* = 0.02) favored the attachment- or relationship-based intervention group.

#### 3.2.6. Effects of Interventions on Maternal Depression

Two studies [22,26] assessed maternal depression using the Edinburgh Postnatal Depression Scale. A homogeneity test revealed Q (chi-square) = 0.51, df = 1 (*p* = 0.48), and I2 = 0%. The pooled effect sizes for maternal depression (MD = −0.64, 95% CI = −0.83 to −0.44, *p* < 0.00001) favored the attachment- or relationship-based intervention group.

#### 3.2.7. Effects of Interventions on Infants’ Weight Growth

Two studies [20,30] measured infants’ weight growth outcomes, with both calculating the velocity of weight gained. Upon examining these two studies—which reported infants’ weight growth in the experimental and comparison groups participating in the attachment- and relationship-based programs—a homogeneity test revealed Q (chi-square) = 0.10, df = 1 (*p* = 0.75), and I2 = 0%. The effect size of infant weight growth was 5.29 (95% CI: 1.96–8.63), and the difference in infant weight growth between the two groups was statistically significant (Z = 3.11, *p* = 0.002).

#### 3.2.8. Effects of Interventions on Infants’ Development

Three studies [18,19,31] reported infants’ developmental outcomes among the intervention and comparison groups participating in attachment- or relationship-based programs. A homogeneity test revealed Q (chi-square) = 4.79, df = 2 (*p* = 0.09), and I2 = 58%. Two studies [18,19] used a mental development index from the Bayley Scales of Infant Development, namely the second and third editions, respectively. Twohig et al. [31] used the Ages and Stages Questionnaire—Social-Emotional Development Version to measure infants’ social, emotional, and regulatory development. The effect size of infants’ development was 0.37 (95% CI = 0.08 to 0.65), with a statistically significant difference observed between the experimental and control groups (Z = 2.53, *p* = 0.01).

### 3.3. Publication Bias

Tests for funnel plot asymmetry and the test proposed by Lee [33] and Sterne et al. [34] should be considered when the meta-analysis includes more than 10 studies because the test power is normally too low to validate chance from true asymmetry [34]. Although a small number of heterogeneous studies with different outcome instruments might limit the analysis, the funnel plot was used to illustrate some asymmetrical distribution shapes.

## 4. Discussion

A systematic review and meta-analysis were performed to investigate the effects of attachment- and relationship-based interventions on attachment/bonding relationship-related, parental-psychosocial-related, or infant health-related outcomes in preterm infants and their families. The systematic literature review provided 15 RCT studies that were conducted from 2001 to 2021 in the United States; United Kingdom; Australia; Europe, or specifically, the Netherlands, Sweden, Switzerland, and Ireland; and Asia, or specifically, Taiwan, India, and South Korea. All studies included mother–preterm infant pairs or dyads and encouraged parents to interact with their infants or involve behavioral reactions in responding to their infants’ cues. The most commonly included component was the mother–infant interaction; 12 of the 15 RCT studies provided programs that combined some form of parent education based on parent–infant interactive behavior and/or parental support. Based on an evaluation of the quality of the literature, it was unclear to the researchers after demonstrating concealment in the allocation sequence as to the intervention/control group among participants. Further, blinding in the intervention group was difficult, as not all NICU staff participants were in the same NICU, and care deliverers knew their group assignments in advance. Therefore, future RCT studies should be designed to address this aspect and improve the quality of such literature.

The results indicated that attachment- and relationship-based interventions for parent–infant dyads in the NICU significantly affected traumatic stress, depression in mothers of preterm infants, and infant weight growth and development. Subgroup analyses suggested attachment- and relationship-based interventions had significantly improved effects on mothers’ interactive controlling or responsive behaviors and children’s interactive behaviors, including compliance, difficulty, and passivity, compared with typical or standard care.

Maternal postpartum attachment was gauged by positive emotions in interactions with the infant, the perception of the caregiving role as a smaller burden, and the quality of mother–infant interactions; such attachment is critical for infants’ optimal growth and development [8,9]. However, the four interventions had no overall effects on parent-to-infant attachment or parental bonding; two studies without parent education components, but the same outcome measure of attachment scale (MPAS), had no significant effects. Various early parenting interventions to address attachment patterns in children with a mean age of less than 13 years were found to be effective in promoting secure attachments [11], while parent–infant interventions for parents with preterm infants during NICU hospitalization had an insignificant effect on parental sensitivity [35]. Our results might not be due to the interventions’ ineffectiveness, but rather their complicated attributes of attachment and heterogeneity (e.g., the time of assessment or duration), despite the intention to improve attachment or bonding between mothers and their preterm infants.

Mother–infant interactions were measured using the CARE-Index in two studies. The test for the interventions’ overall effects on the quality of mother–infant interactions was not significant. Regarding the subgroup analysis according to the intervention/program’s target outcome (e.g., subscales of mother–infant interaction), mothers’ and children’s interactive behaviors were classified into their dimensional categories. In terms of effect size, intervention studies focused on children’s interactive behaviors, such as infant difficulty (1.26) and passivity (−0.68), while mothers exhibited relatively larger interactive controlling behaviors (−0.60). A significant difference was observed between the two groups classified by the subscales of mother–infant interactions, with the interactions’ effect size noted according to the sub-dimensions of mothers’ and children’s interactive behaviors. While the CARE-Index examines patterns of mother–infant interactions by rating the interactive behaviors (e.g., facial or vocal expressions, position, physical contact, and expressions of affection) and correlations that exist between the mother and infant dimensions—for example, controlling and compliant, or unresponsive and passive [36], our results might reflect the CARE-Index tool’s characteristics. It would be beneficial for clinicians or health providers to consider the weak dimensions of mother–infant interactions when identifying at-risk parent–infant dyads and designing NICU family interventions for them. Moreover, very few studies in the systematic review selected for this study measured mother–infant interactions as an outcome variable; thus, future studies must investigate the effects of attachment- and relationship-based interventions on the parent–infant relationship and interactions among the NICU population.

Regarding parental psychosocial outcomes, the attachment- and relationship-based interventions implemented for preterm infants and their families are significantly effective in reducing maternal traumatic stress (SMD = −0.33) and depression (MD = −0.64). In a previously conducted meta-analysis that assessed the effect of NICU-based intervention/family-centered care on maternal depressive or anxiety symptoms, such programs were found to alleviate parental anxiety, stress, and depression [37,38], similar to our results. Maternal mental health, as indicated by higher levels of depression in mothers of preterm infants, affects the poorer quality of attachment relationships [9]. Moreover, psycho-emotional distress (i.e., post-traumatic stress or mood disorders) is linked with postpartum maternal attachment to the infant, as illustrated by the mother’s representation of herself and the infant after preterm birth [8]. Therefore, it is necessary to provide an attachment- and relationship-based intervention that includes parental support (i.e., counseling, consulting sessions, or discussions) tailored to a psychosocially vulnerable parent’s circumstances and psychological concerns.

This study’s results demonstrated that attachment- and relationship-based interventions significantly affect infants’ weight growth and development. Our results partially correspond with prior research based on English and Chinese databases [37], which suggested that NICU interventions related to family-centered care of preterm infants and their parents positively impacted infants’ weight gain; however, no statistical differences were observed in their neurobehavioral development. These findings are consistent with those of Burke [39], in that developmental care in the NICU had some positive effects on preterm infants’ neurodevelopment outcomes. Our findings reinforce the importance of attachment- and relationship-based nursing interventions for parent–infant dyads in the NICU as a key to infants’ growth and development.

Meanwhile, this systematic review’s causality is limited because few RCT studies have performed attachment- and relationship-based interventions during infant hospitalization in NICUs. The generalizability would be restrictive, as we included only English literature, and meta-analyses were conducted with fewer than 10 studies, indicating possible lower test power. Further, future research should focus on measuring mother–infant interactions, attachment- and relationship-related outcomes, and parental psychosocial and infant growth and development at standardized time points in the same gestational age group.

Based on this study’s results, components of attachment- and relationship-based interventions (mother–infant interaction, parent education, support) should be operationalized consistently in diverse international settings. Further, greater effort is needed to not only ensure program fidelity, but also collect more objective electronic medical data (e.g., bio-behavioral, physiological, or electroencephalogram-based information), as “real world” data [40] in conjunction with a multidisciplinary team. Relying only on self-reported data can under- or overestimate program effectiveness, so it is thought that the evidence for program effectiveness will be stronger if objective evaluation data are used.

## 5. Conclusions

The present study examines the effects of attachment- and relationship-based interventions on relieving maternal traumatic stress and depression to facilitate mother–child interactive behaviors, infant weight gain, and development in preterm infants. The effects of a NICU intervention can improve when it includes key components of parent–infant interactions; parent education, such as the information given, guided observations, and active involvement; support or counseling; and consulting tailored to parents’ circumstances and psychological concerns. High-quality, staged trials—including those to analyze early/initial neonatal hospitalizations as well as the transition from hospital to home—based on mother–infant attachments/bonding and relationships in the NICU are still needed to robustly establish the effects of such tailored interventions on sustainable, long-term, and important outcomes.

## Figures and Tables

**Table 1:** Characteristics and methods of studies included in the review (*n* = 15).

First Author (Year)	Risk of Bias	Country of Study	No. of Centers (*n*)	Infants Enrolled (*n*)	Group Allocation		Mean Gestational Age (Weeks)		Mean Birth Weight (Grams)		Follow Up
					I ^a^/I ^b^	C/R ^c^	I ^a^/I ^b^	C/R ^c^	I ^a^/I ^b^	C/R ^c^	
Melnyk et al. (2001) [19]	Low	United States	1	42	20	22	31.4	31.6	1482.7	1731.0	
Ramanathan Paul, Deorari, Taneja, and George (2001) [20]	Low	India	1	24	14	14	(median) 30.4	(median) 30.9	1219.0	1270.9	
Browne and Talmi (2005) [17]	Low	United States	1	84	28 ^a^/31 ^b^	25	32.0 ^a^/31.2 ^b^	31	1617.4 ^a^/ 1509.3 ^b^	1518.0	
Lai et al. (2006) [21]	Low	Taiwan	2	30	15	15	33.8		2248		
Miles, Cowan, Glover, Stevenson, and Modi (2006) [22]	Low	United Kingdom	2	78	42	32	28	28	1086	1133	
Schroeder and Pridham (2006) [23]	Some concerns	United States	2	16	8	8	26.8	27.5	828	1049	
Glazebrook et al. (2007) [24]	High	United Kingdom	6	210	99	111	20	21	(median) 1120	(median) 1220	
Borghini et al. (2014) [25]	Low	Switzerland	1	83	30	30/23 ^c^	30	30/39 ^c^	1343	1435/3281	12 months CA
Wu et al. (2014) [18]	Low	Taiwan	3	178	57 ^a^/63 ^b^	58	30.0 ^a^/29.9 ^b^	29.3	1179 ^a^/1149 ^b^	1091	24 months
Hoffenkamp et al. (2015) [26]	Low	Netherlands	7	150	75	75	32	32	1828	1770	at 1, 3, and 6 months postpartum
Mörelius, Örtenstrand, Theodorsson, and Frostell (2015) [27]	Low	Sweden	2	42	23	19	34	34	2468	2512	at 1 and 4 months CA
Samra et al. (2015) [28]	Low	United States	1	40	20	20	35	35.5	2493	2693.8	at discharge
Evans, Boyd, Colditz, Sanders, and Whittingham (2017) [29]	Low	Australia	2	145	75	70	28.51	28.55	1159.3	1107.8	at 12 months CA
Heo and Oh (2019) [30]	Some concerns	South Korea	1	66	33	33	28.42	29.75	1139.6	1228.1	
Twohig et al. (2021) [31]	Some concerns	Ireland	1	80	42	38	28.4	28.3	1179	1176	at 6,9, and 12 months CA

Note. C: control group; CA: corrected age; I: intervention group (I ^a^/I ^b^ = 1st Intervention Group/2nd Intervention Group); R ^c^: reference group (full-term infants).

## Data Availability

No new data were created or analyzed in this study. Data sharing is not applicable to this article.

## Supplementary Materials

The following supporting information can be downloaded at: https://www.mdpi.com/article/10.3390/ijerph19031126/s1, Figure S1: Forest plot of comparisons; Table S1: Intervention components, coded categories, and attachment/relationship, parental psycho-social, and infant outcomes; Table S2: Synthesis of results with the 8 variables.
