# Supplementary material for: Attachment- and Relationship-Based Interventions during NICU Hospitalization for Families with Preterm/Low-Birth Weight Infants: A Systematic Review of RCT Data
Source: Int J Environ Res Public Health. 2022 Jan 20;19(3):1126. doi: 10.3390/ijerph19031126 (PMC8834457; doi:10.3390/ijerph19031126)
Supplement: Supplementary file 1 [file ijerph-19-01126-s001.zip › ijerph-1523898-supplementary.pdf]

Supp1.

Table S1. Intervention components, coded categories, and attachment/relationship, parental psycho-social, and infant outcomes (N = 15).

First Author (Year)	Name of Intervention	Who Delivered	Duration/ Number of Sessions	Control or Comparison Group	Intervention Components					Attachment/ Relationship Outcomes	Parental Psychosocial Outcomes	Infant health outcomes
					Parent–Infant Interaction	Parent Education E1	E2	E3	Support			
Melnyk et al. (2001)[19]	Creating Opportunities for Parent Empowerment (COPE)	n.a.	4-phase (6-month)	4-phase comparison program	+	+		+		- (maternal-infant interaction scale /MIIS)	- (state anxiety/STAI; negative mood state/POMS); + (sights and sounds stress related to NICU/PSS:NICU)	3 & 6 months' corrected age + (Infant cognitive development / MDI)
Ramanathan et al. (2001)[20]	Kangaroo Mother Care (KMC)	Mother & nurse	4 hours/day	Standard care	+					n.a.	6-week follow-up + (no. of infants	+ (weight gain;

									exclusively breastfeeding)	age at hospital discharge)
Browne & Talmi (2005)[17]	G1 (Demonstration & Interaction)<br><br>G2 (Education)	n.a.	45 minutes	Informal<br><br>discussion	+	+	+	+<br><br>(feeding interaction of both parent and infant/NCAFS)	1 month after discharge G1 & G2 < control +<br><br>(stress related to child rearing/PSI)	n.a.
Lai et al. (2006)[21]	Music during Kangaroo care	Mother & nurse	60 minutes,<br><br>3 days	Routine care	+			n.a.	+<br><br>(maternal state anxiety/STAI)	-<br><br>(physiological parameters / oxygen saturation, heart rates, respiratory rates)<br><br>+<br><br>(behavioral state: quiet sleep, crying, active awake)

Miles et al. (2006)[22]	mother-infant Skin-to-Skin contact (STS)	Mother & nurse	20 minutes/day, 4 weeks	Standard care	+				- (parent to infant attachment /MPAS)	- (maternal stress/PSS:NICU; Perception of own caregiving confidence/MABS; Depressive symptoms/EPDS; Anxiety levels/STAI; Mental health/GHQ-28; dysfunctional parental behavior and stressors in the parenting relationship/parent domain-PSI)	-
Schroeder and Pridham (2006)[23]	Guided Participation (GP)	Research nurse/expert practitioner	45 minutes, 1 session / 6 weekly	Standard care education	+	+	+	+	+ (mother's expectations and intentions	n.a.	n.a.

										concerning relationship/ WMRS; relationship competencies/ RCA)		
Glazebrook et al. (2007)[24]	Parent Baby Interaction Program (PBIP)	Nurse	1-hour, weekly	Control phases	+	+	+			-<br>(caregiver–child interaction<br>/NCATS)	-<br>(stress in the parent-child system/PSI-SF)	-<br>(newborn competence in seven functional domains/NAPI)
Borghini et al. (2014)[25]	Intervention program	Nurse & therapist	Until 4 months corrected age	Without intervention<br>(no interaction guidance)	+		+	+		-<br>(quality of mother-infant interactions/<br>CARE-Index)	+<br>(maternal posttraumatic stress symptoms/<br>PPQ)	n.a.
Wu et al. (2014)[18]	Clinic-Based Intervention Program (CBIP)	Nurse &<br>physical therapist	*(5 sessions in hospital)*	G1: Home-Based Intervention Program (HBIP)	+	+			+	+<br>(mother–infant interaction/<br>free-play procedure)	n.a.	24 months+<br>(infant developmental outcomes/<br>(Bayley-III)

				G2: Usual Care Program (UCP)						- (infant behavioral outcomes /CBCL)
Hoffenkamp et al. (2015)[26]	hospital-based Video Interaction Guidance (VIG)	certified VIG professional (nurse and pedagogic workers)	Daily (15 minutes/day)	Standard hospital care	+	+	+	+ (parental interactive behavior; maternal & paternal bonding/ PBQ, MBI, YIPTA)	- (parental stress/PSS:NICU; depressive symptoms/EPDS; maternal trauma/TES)	n.a.
Mörelius et al. (2015)[27]	Skin-to-Skin Contact (SSC)	Parents/ nurse	24 hours/day until hospital discharge	Standard care	+	+		n.a.	+ (mothers' & fathers' parenthood stress/SPSQ) - (salivary cortisol;	+ (lower salivary cortisol reactivity)

									mothers' depression/EPDS; parental sensitivity/Ainsworth's sensitivity scale)	
Samra et al. (2015)[28]	Skin-to-Skin Care (SSC)	Mother/ bedside nurse	50 minutes (3 times a week)	Standard care (hold SSC)	+	+		n.a.	+ (maternal stress/ PSS:NICU)	n.a.
Evans et al. (2017)[29]	Baby Triple P (BTP)	Facilitator	4 sessions (in hospital, 2 hours/session); 4 sessions (at home, 30 minutes/session)	Care as Usual (CAU)	+	+	+	- (mother–infant relationship/ EA; maternal postnatal attachment /MPAS; - maternal responsiveness to infant	n.a.	n.a.

										cues/MIRI)		
Heo and Oh (2019)[30]	Parent Participation Improvement Program	Nurses & mother-father dyads	2-week program	Routine visits	+	+	+	+		+ (maternal & paternal attachment /MAI)	+ (parents' partnerships with nurses/Pediatric Nurse-Parent Partnership Scale)	- (body weight)
Twohig et al. (2021)[31]	Preterm Infant-Parent Program for Attachment (PIPPA)	Clinician (child psychiatrist)	3 hours *(3 sessions)*	Standard care	+	+	+	+	+	9 months- (patterns of interactions of infants and caregivers/ CARE-Index)	n.a	12 months+ (infant social, emotional, and regulatory development /ASQ-SE)

Notes: +/- = significant effect/no significant effect; ASQ-SE = Ages and Stages Questionnaire, Social-Emotional Development version; Bayley-II = Bayley Scales of Infant and Toddler Development, 3rd ed.; CARE = The Child Adult Relationship Evaluation; CBCL = Child Behavior Checklist for Ages 1.5 to 5; EA = Emotional Availability Scale; EPDS = Edinburgh Postnatal Depression Scale; GHQ-28 = General Health Questionnaire; MABS = Mother and Baby Scale; MAI = Maternal Attachment Inventory; MBI = My Baby and I Questionnaire; MDI = Mental Development Index of the BSID-II (Bayley Scales of Infant Development, 2nd ed.); MIIS = Maternal-Infant Interaction Scale; MIRI = Maternal Infant Responsiveness Instrument; MPAS = Maternal Postnatal Attachment Scale; n.a. = not assessed or not reported; NAPI = Neurobehavioral Assessment of the Preterm Infant; NCAFS = Nursing Child Assessment Feeding Scale; NCATS = Nursing Child Assessment Teaching Scale; PBQ = Postpartum Bonding Questionnaire; POMS = Profile of Mood States; PPQ = Perinatal Posttraumatic Stress Disorder Questionnaire; PSI = Parenting Stress Index; PSI-SF = Parenting Stress Index, short form; PSS:NICU = Parental Stressor Scale: Neonatal Intensive Care Unit; RCA = Relationship Competencies Assessment; SPSQ = The Swedish Parenthood Stress Questionnaire; STAI = State-Trait, Anxiety Inventory; TES = Traumatic Event Scale; WMRS = Working Model Relationship Score; YIPTA = Yale Inventory of Parental Thoughts and Actions;

Coding categories for parent education intervention components [31]: E1 = information given: includes information that is generic (e.g., written or audio or video recorded), individualized to the family (e.g., written or verbal), and/or includes discussion with the parent; E2 = Guided observation: includes parent observations or demonstrations of an activity with the infant; E3 = Active involvement: includes parent involvement in active practical experiences, modeling and guided self-evaluation or self-reflecting education (e.g., video feedback); Support = includes parent/family support sessions and/or counseling/consulting to support parents (e.g., discussions tailored to a parent's circumstances and addressing any socio-emotional/psychological concerns).

Table S2. Synthesis of results with the 8 variables (N = 10).

First Author (Year)	Attachment/ bonding	Mother-infant interactions	Maternal anxiety	Parental environmental stress	Maternal traumatic stress	Maternal depression	Infant weight growth	Infant development
Melnyk et al. (2001)[19]			+	+				+
Ramanathan et al. (2001)[20]							+	
Miles et al. (2006)[22]			+			+		
Borghini et al. (2014)[25]	+	+		+	+			
Wu et al. (2014)[18]								+
Hoffenkamp et al. (2015)[26]				+	+	+		
Mörelius et al. (2015)[27]								
Samra et al. (2015)[28]				+				
Evans et al. (2017)[29]								

Heo and Oh (2019)[30]							+	
Twohig et al. (2021)[31]	+	+						+

Figure S1. Forest plot of comparisons

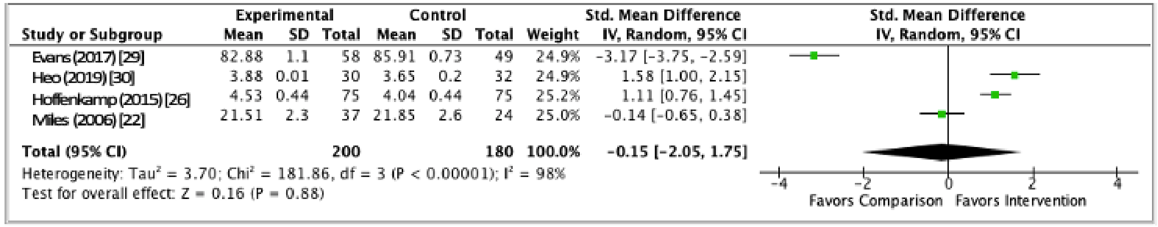

1. Outcome: Parent-to-infant attachment or parental bonding

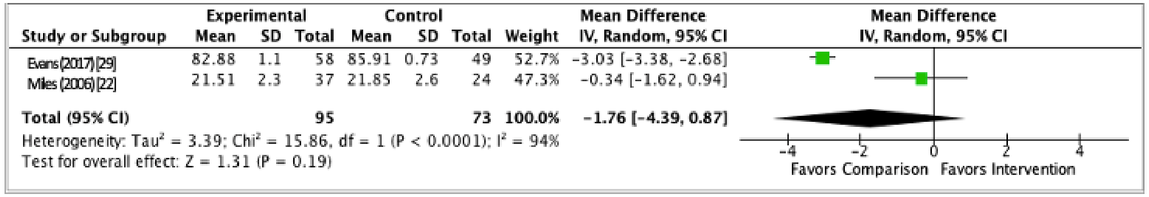

1-1. Outcome: Maternal postnatal attachment scale (sensitivity analysis)

Miles et al. (2005)[22]: Parent-infant interaction

Hoffenkamp et al. (2015)[26]: Parent-infant interaction; parent education (information given and active involvement)

Evans et al. (2017)[29]: Parent-infant interaction; support

Heo and Oh (2019)[30]: Parent-infant interaction; parent education (information given, guided observations, and active involvement)

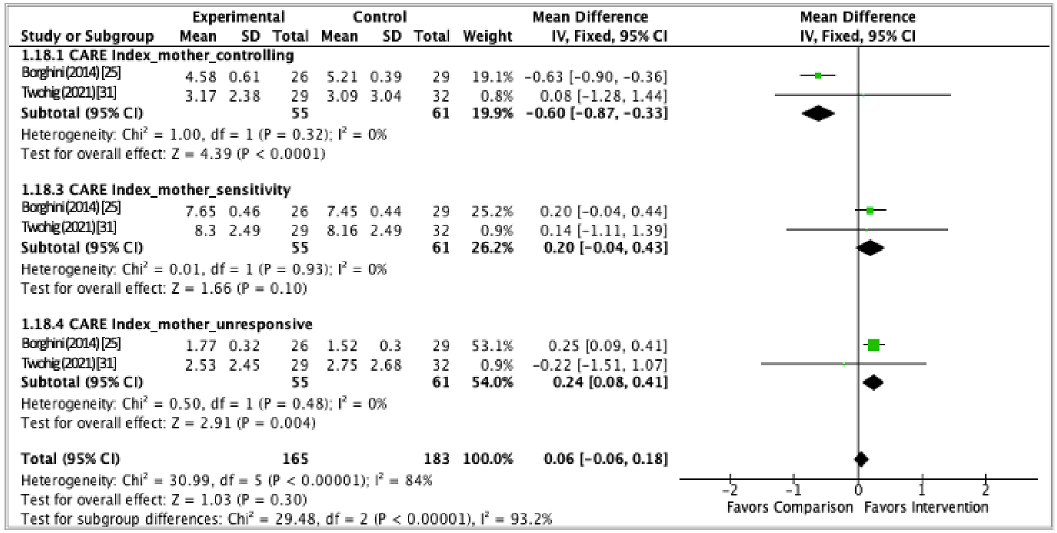

2-1. Outcome: Quality of mother-infant interactions (mother’s interactive behavior)

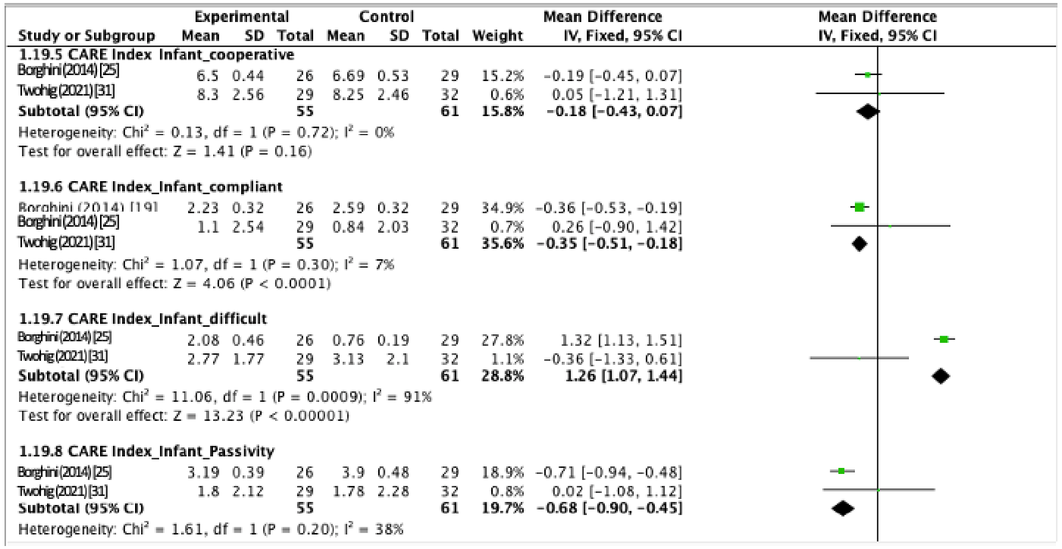

2-2. Outcome: Quality of mother-infant interactions (child’s interactive behavior)

Borghini et al. (2014)[25]: Parent-infant interaction; parent education (guided observation and active involvement)

Twohig et al. (2021)[31]: Parent-infant interaction; parent education (information given, guided observations, and active involvement); support

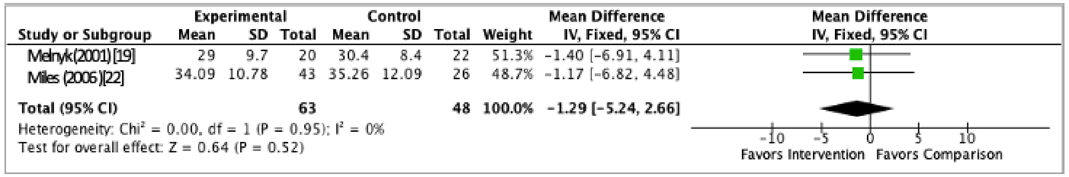

3. Outcome: Maternal anxiety levels

Melnyk et al. (2001)[19]: Parent-infant interaction; parent education (information given, active involvement)

Miles et al. (2006)[22]: Parent-infant interaction

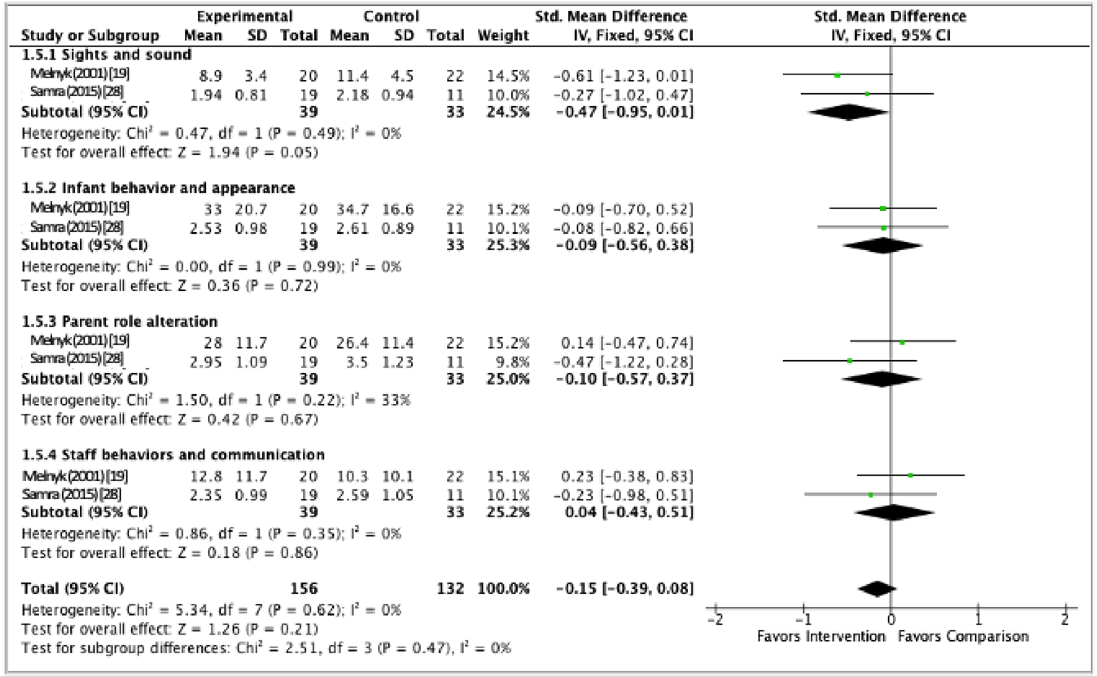

4. Outcome: Parental environmental stress

Melnyk et al. (2001)[19]: Parent-infant interaction; parent education (information given, active involvement)

Samra et al. (2015)[28]: Parent-infant interaction; parent education (information given)

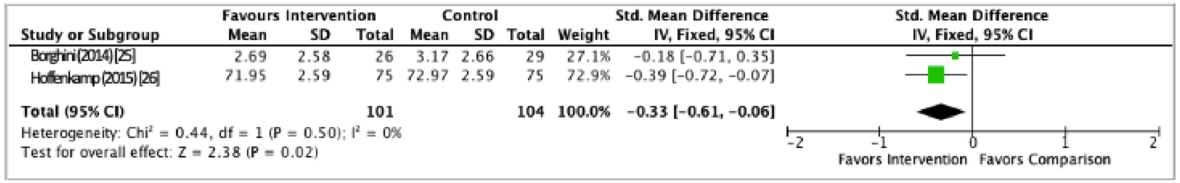

5. Outcome: Maternal traumatic stress

Borghini et al. (2014)[25]: Parent-infant interaction; parent education (guided observation and active involvement)

Hoffenkamp et al. (2015)[26]: Parent-infant interaction; parent education (information given and active involvement)

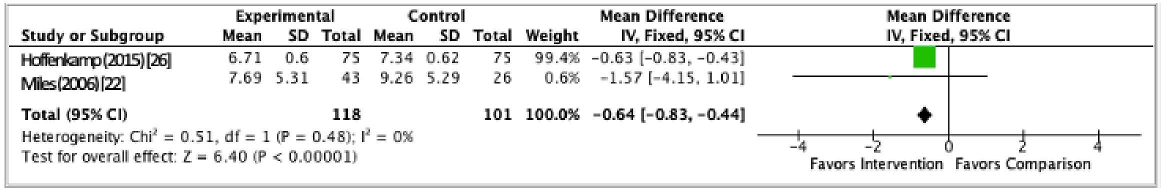

6. Outcome: Maternal depression

Miles et al. (2006)[26]: Parent-infant interaction

Hoffenkamp et al. (2015)[22]: Parent-infant interaction; parent education (information given and active involvement)

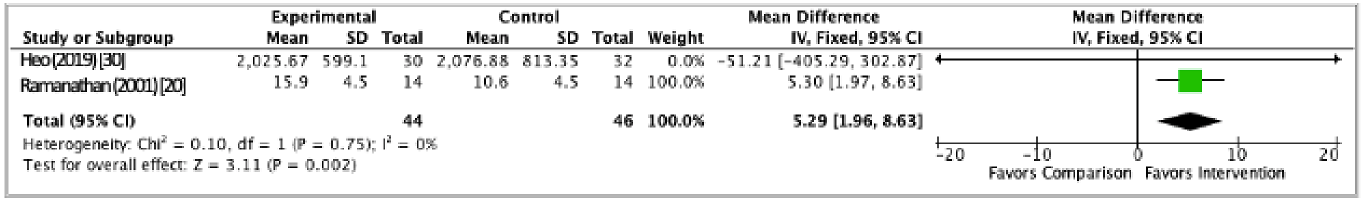

7. Outcome: Infant growth (birth weight)

Heo and Oh (2019)[30]: Parent-infant interaction; parent education (information given, guided observations, and active involvement)

Ramanathan et al. (2001)[20]: Parent-infant interaction

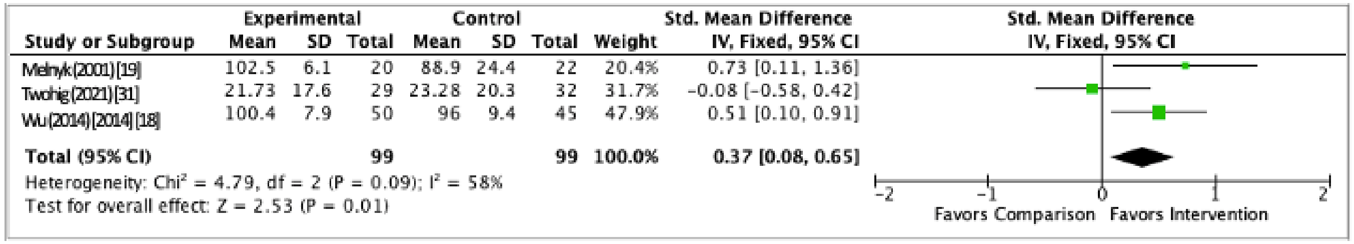

8. Outcome: Infant development

Melnyk et al. (2001)[19]: Parent-infant interaction; parent education (information given and active involvement)

Twohig et al. (2021)[31]: Parent-infant interaction; parent education (information given, guided observations, and active involvement); support

Wu et al. (2014)[18]: Parent-infant interaction; parent education (information given); support

Figure S1. Forest plot of comparisons
